# The dilemma of agricultural pollination in Brazil: Beekeeping growth and insecticide use

**DOI:** 10.1371/journal.pone.0200286

**Published:** 2018-07-06

**Authors:** Charles Fernando dos Santos, Alex Otesbelgue, Betina Blochtein

**Affiliations:** 1 Departamento de Biodiversidade e Ecologia, Escola de Ciências, Pontifícia Universidade Católica do Rio Grande do Sul, Porto Alegre, Rio Grande do Sul, Brazil; 2 Instituto do Meio Ambiente, Pontifícia Universidade Católica do Rio Grande do Sul, Porto Alegre, Rio Grande do Sul, Brazil; University of California San Diego, UNITED STATES

## Abstract

Pollination by bees improves agricultural crop yields and improves the financial outlook of beekeepers because it increases honey production and hive rental revenues. However, in Brazil, with a few exceptions, these benefits have been neglected in recent years because beekeepers are more interested in honey production than in agricultural pollination. The excessive and indiscriminate use of insecticides on agricultural fields in Brazil appears to be one of the principal obstacles preventing partnership between farmers and beekeepers. The goal of this study was therefore to evaluate the most recent situation in Brazil in relation to the use of insecticides, agriculture and to honey production in comparison with other countries. Our results show that Brazil is the largest consumer of insecticides in the world and that consumption has increased by > 150% over 15 years. While countries with a high Human Development Index (i.e., a measure that can also be used to question national policy choices) are reducing their levels of insecticide use in agriculture, Brazil is going in the opposite direction. It is highly likely the increase seen in other countries is a result of alternative methods for pest control rather than a result of the amount of area under agricultural cultivation and their capability to shift their economies from agriculture to other sectors. The number of hives (23%) and the volume of honey production (72%) in Brazil have, however, increased over the same period, raising Brazil to the ninth highest honey producer in the world. Although the data on apiculture are promising, the growth in use of insecticides in Brazil is a cause for concern because they leave residuals on bee products, on crops, and in the environment. Civil society and government in Brazil should encourage reductions in insecticide use and better relations between agricultural farmers and beekeepers.

## Introduction

Bees contribute to the cross-pollination of many plant species [1]. These insects play an important role in agricultural systems as agents of pollination, thereby contributing to improving the yields of crops [2]. Despite this, it is known that we are currently facing a global pollination crisis [3]. This crisis threatens a deficit of pollinators (e.g., bees) able to meet the demands of agricultural crops [4]. Recent works have demonstrated that biodiversity loss related to functional diversity of organisms may have deep consequences for ecosystem resilience because functional groups providing valuable ecological services, such as pest control and pollination, have declined [5,6]

Brazil is a world leader in agricultural production, and many of its agricultural crops depend to a lesser or greater degree on the pollination services provided by bees [7–9]. Studies have estimated that the economic value of crop pollination services provided by bees in Brazil is approximately $12–14 billion [8,10]. Despite the elevated value attributed to bees, with a few exceptions, keepers of honey bees and stingless bees in Brazil invest greater effort in honey production than in renting their hives for agricultural pollination [11–13].

Recent data show that Brazilian honey production came close to 40,000 metric tons, with profits of around $82 million [14]. Thus, while honey production is the main concern of Brazilian beekeepers, the use of bees for agricultural pollination has been relatively neglected [8,10]. Crop pollination may be beneficial to beekeepers, increasing their income [15–17]. Similarly, it would also contribute to increasing the revenues from Brazilian harvests because both the quantity and quality of fruit and seeds are increased when pollination is performed by bees, which increases their market value [17–20].

Considering that both industries could increase their profits through agricultural pollination services provided by bees, it might be expected that there would be a closer partnership between agricultural farmers and beekeepers in Brazil. However, excessive use of insecticides on Brazilian agricultural crops [21–23] could be detrimental for bees. For example, in a recent case study, 90% of interviewed beekeepers reported beehive loss because of agrochemicals being used on crops close to their apiaries [24].

Although bee mortality caused by the lethal effects of agrochemicals garners more visibility by the general public or media due to its dramatic impact [24], the less-detectable sublethal effects of agrochemicals are also seriously detrimental to the long-term survival and viability of hives [25,26]. For example, field studies have detected a plethora of insecticides (e.g., neonicotinoids, fipronil) at residual levels in brood, pollen, honey, and wax, which may compromise the growth, strength, and survival of the bee hives [27,28]. Many experimental studies have shown that insecticides compromise homing, memory abilities, cognition, foraging, and navigation in workers [25–27,29,30], sperm motility in drones [31], and emergence, survival, and reproduction of queens [32–34]. Furthermore, as insecticides act synergistically with other contaminants (e.g., other insecticide classes, fungicides), pathogens (e.g., *Nosema ceranea*), and nutritional stressors, their negative effects on bee behavior, physiology, and immunity are even more dramatic [27,35–40]. Human health may also be indirectly affected; honey sold at supermarkets worldwide has been found to be contaminated with residual levels of neonicotinoids, demonstrating their long-term presence within colonies [41].

There is no doubt that taking advantage of the benefits offered by bees while at the same time controlling the thousands of agricultural pests using insecticides is a challenge. Brazilian agriculture is faced with approximately 560 species of agricultural pest insects that are controlled mostly with insecticides [21–23]. As such, a proportion of these pesticides have low toxicity to bees and the environment [42] (S1 Fig). This practice can have direct or indirect effects on the 3,000 or so species of wild bees [43] in Brazil and can be harmful to the approximately 1 million hives of honey bees [14] and ~18,000 hives of stingless bees [13] that could be used for agricultural pollination. In other words, all this potential that bees offer is being systematically ignored by Brazilian agriculture for the pollination of its crops.

Pest insects cause large productivity losses and consequently have a major economic impact on Brazilian agriculture [44]. However, it is necessary to find a compromise that is not detrimental to the benefits of pollination by bees through use of sustainable alternatives, since agriculture and apiculture/meliponiculture (stingless beekeeping) are complementary activities rather than rivals. Thus, different agricultural activities may be adopted as alternative or complementary practices to agrochemical pest control [45]. For example, methods such as semiochemical control, biological control (predators, parasitoids), entomopathogenic fungi, botanical insecticides, essential oils, crop rotation, recombinant RNA technologies, and even organic cultivation practices can be combined into integrated pest management (IPM) strategies [45–49].

It is known that approximately 60% of Brazilian crops depend primarily on bees for pollination [8]. However, while bee management in fields could both benefit honey production [15–17] and raise crop yields [17–20], increasing insecticide use in Brazil [21–23] could hamper this business [11]. Furthermore, the increase in Brazilian agricultural productivity due to pesticide (e.g., insecticide) use has other costs, such as risks to human health, and poses a great challenge for the preservation of environmental quality [21]. Our objective was to evaluate the Brazilian situation over the last 15 years by analyzing indicators related to use of insecticides, honey production and number of hives and, where possible, to compare Brazil’s position in relation to these issues with other countries. The approach taken was to assess these data in the light of potential conflicts of interest between agricultural farmers and beekeepers in Brazil, with the objective of proposing viable options and reliable solutions.

## Materials and methods

We collected data for our analyses from the United Nations Food and Agriculture Organization (http://www.fao.org/faostat/en/#data) [14]: honey production, number of beehives, insecticide use, agricultural area of Brazil and, where needed, other selected nations. Such data can also be freely obtained from national publications, web sites, or trade files as well as from official publications from individual countries [14]. We surveyed for Livestock Primary (**item**: honey; **elements**: production quantity), Live Animals (**item**: beehives; **elements**: stocks), Pesticide Use (**item**: insecticides+Total; **elements**: use) and Land Use (**item**: agricultural area, organic agricultural area; **elements**: area).

**Fig 1:**
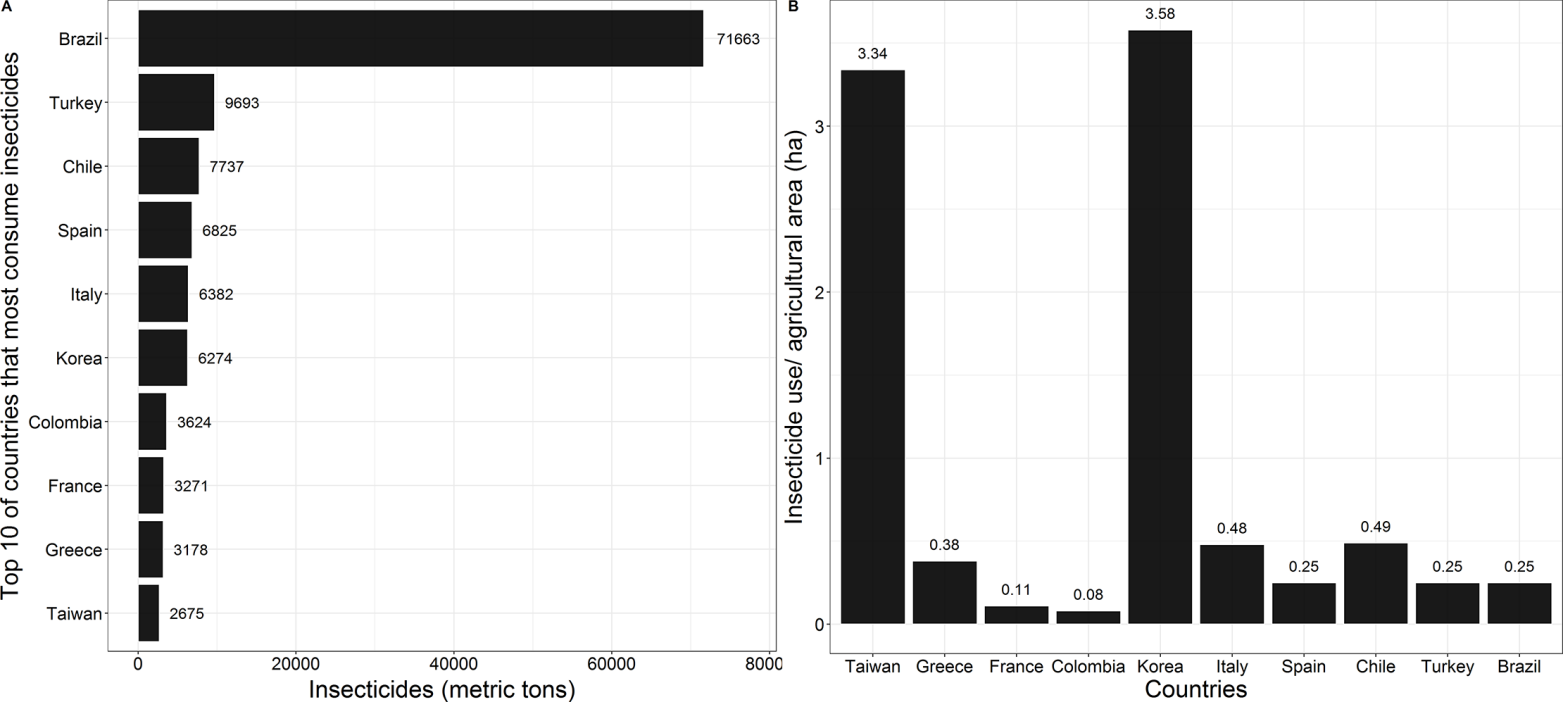
Insecticide use. **A**–World’s 10 leading consumers of active ingredients of insecticides (in metric tons), reference year 2015. **B**–Insecticide use divided by total of agricultural area. Data adapted from the United Nations Food and Agriculture Organization [12]. FAOSTAT search query: Pesticide Use (**item**: *Insecticides + Total*, **elements**: *use*).

Our first step was to rank Brazil among other countries in terms of insecticide use, honey production, total agricultural area, and organic agricultural area. To achieve this, we compiled data on the most recent reference year (2015) related to the quantity of active ingredients for insecticides (in metric tons) and ranked the 10 countries (of the 39 for which data were available) that were the greatest consumers of these agricultural supplies (Fig 1). Information for the reference year was missing from many countries, such as the United States, for which the most up-to-date official figures on the use of insecticides are from 2012 (66,770 metric tons of active ingredients). Therefore, we are aware that these missing values could distort the current country rankings (for more details, see S1 Table). We also plotted the insecticide use of these countries divided by agricultural area (ha) to visualize how these nations have effectively managed their croplands concerning insecticide use. Similarly, we performed the same procedure for honey production.

### Data analysis

**Fig 2:**
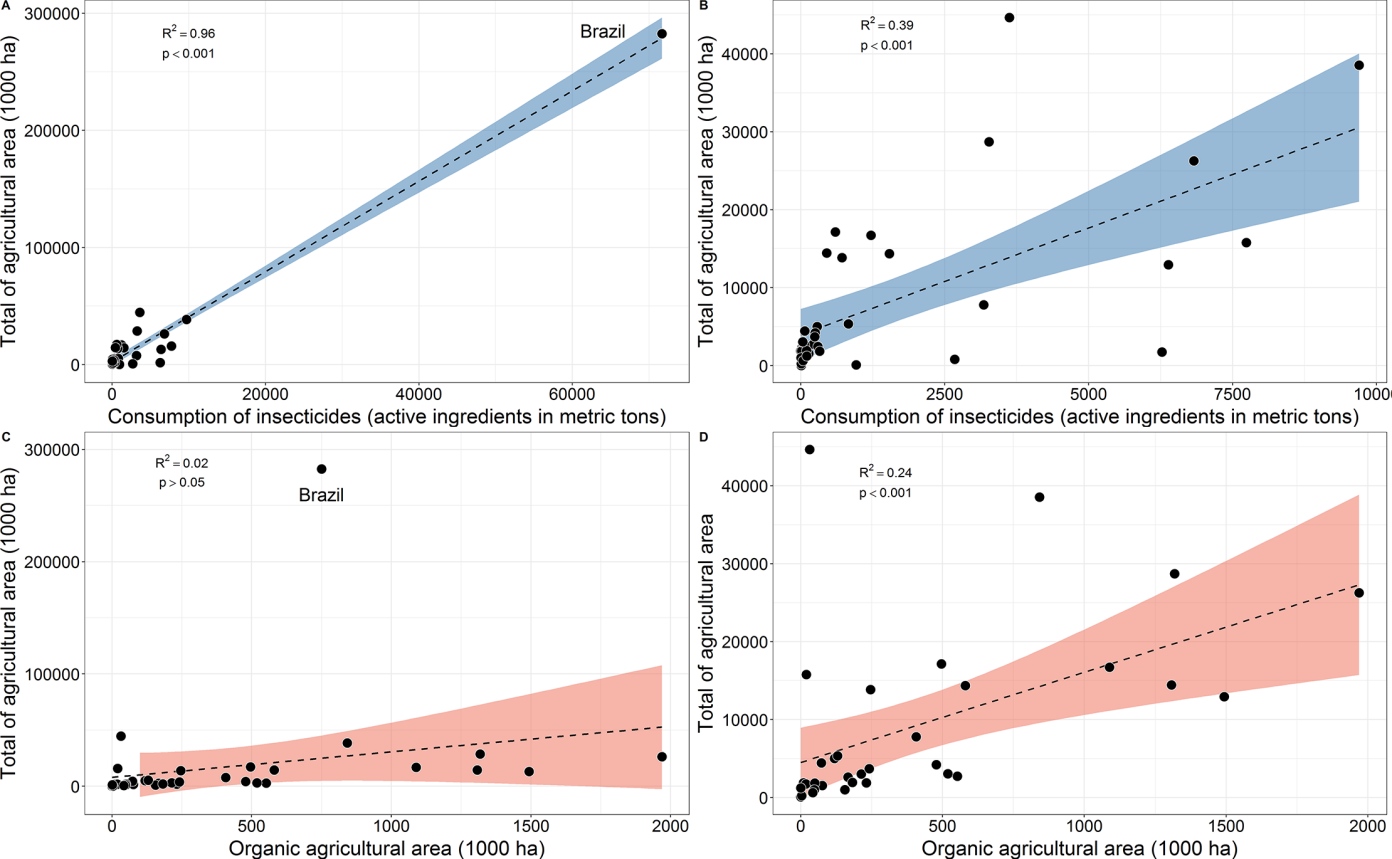
Land area (in 1000 ha) allocated to agriculture for all countries (n = 39) with available data on consumption of active ingredients of insecticides (in metric tons) in 2015. **A**–Insecticide use vs. total agricultural area, with Brazil; **B–**Insecticide use vs. total agricultural area, without Brazil; **C**–Total agricultural area vs. area allocated to organic agriculture, with Brazil; **D**–Total agricultural area vs. area allocated to organic agriculture, without Brazil. For additional details, see S3 Table. Data adapted from the United Nations Food and Agriculture Organization [12]. FAOSTAT search query: Land Use (**item**: *country area*, *agricultural area*, *agricultural area organic*, **elements**: *area*).

**Fig 3:**
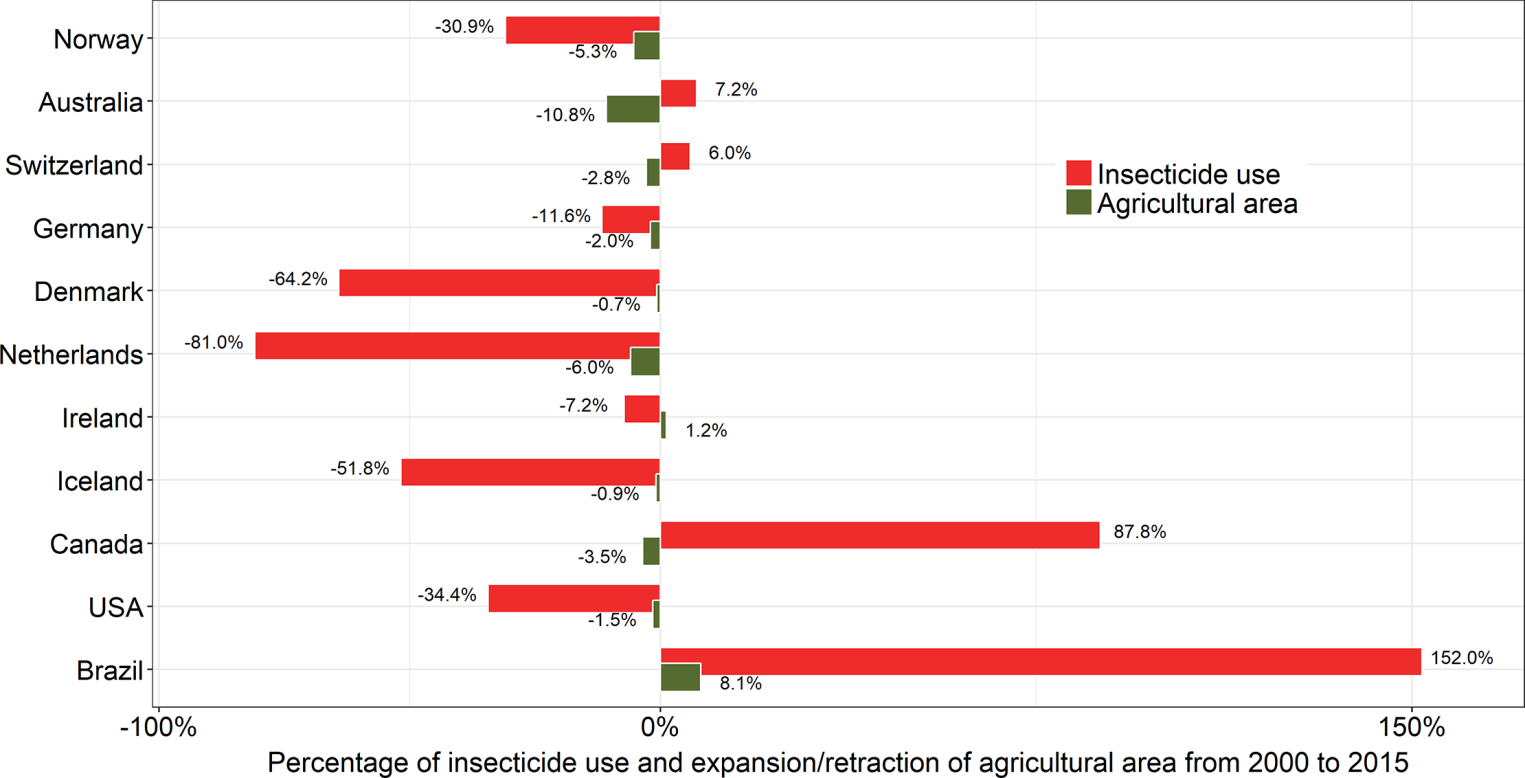
Insecticide use and expansion/retraction of land use for agriculture over the last 15 years among the top-10 ranked countries (decreasing order) in terms of human development index (HDI) and Brazil (ranked 79^th^). Here, HDI was used as a summary measure of average quality of the nations. This measure emphasizes that people and their capabilities should be the ultimate criteria for assessing the development of a country, not economic growth alone. The HDI can also be used to question national policy choices. For more details, see http://hdr.undp.org/en/data or http://hdr.undp.org/en/content/human-development-index-hdi. Data adapted from the United Nations Food and Agriculture Organization [12].

**Fig 4:**
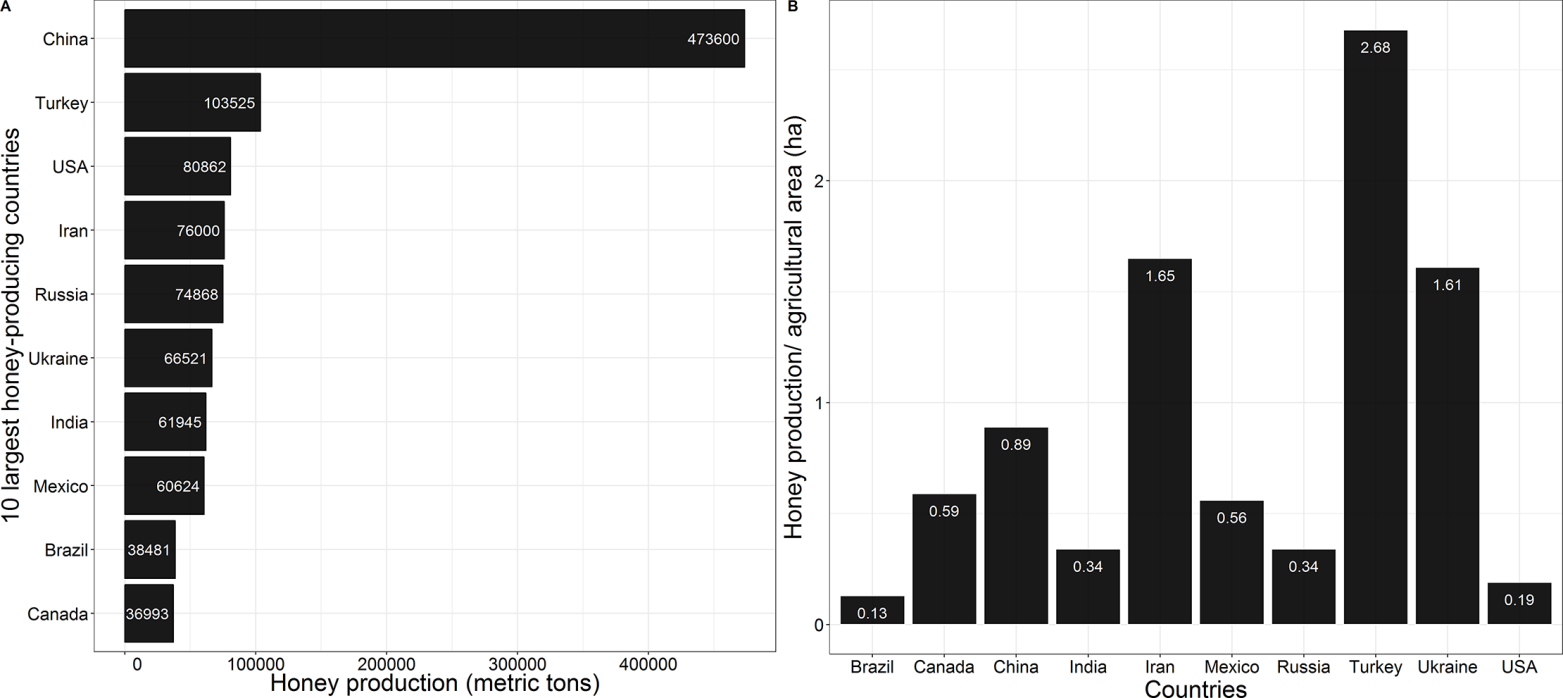
Honey production. **A**–World’s 10 leading honey producers, reference year 2015. **B**–Honey production divided by total of agricultural area. Data adapted from the United Nations Food and Agriculture Organization [12]. FAOSTAT search query: Livestock Primary (**items**: *honey*; **elements**: *production quantity*).

We performed a linear regression analysis using the *lm* function to test whether there was any relationship between use of insecticides and the size of total agricultural area, as well as the latter variable vs. size of organic agricultural area of all countries (*n* = 39), with available data on insecticide use in 2015; see Fig 2A–2D. We were also interested in observing the top-10 ranked countries in terms of the human development index (most up-to-date HDI of 188 available countries) and comparing them with Brazil (ranked 79^th^) to illustrate the amount of insecticide use and expansion/retraction of land used for agriculture over the last 15 years (Fig 3). As part of these analyses comparing Brazil with other countries, we plotted the 10 (of ~165) largest honey-producing countries to determine where Brazil ranks according to the most recent data (2015) (Fig 4).

**Fig 5:**
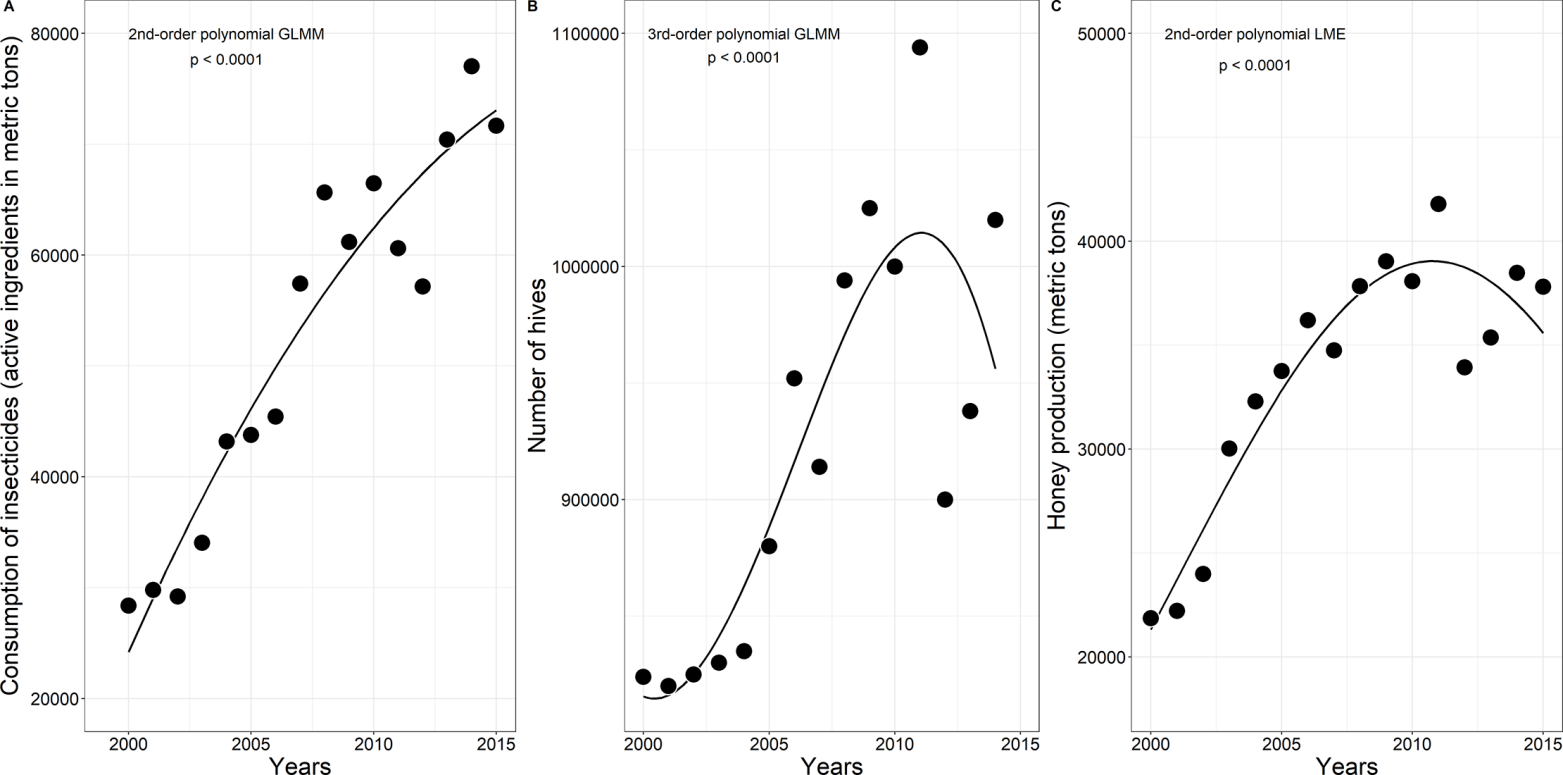
**A) Use of active ingredients of insecticides; B) Number of beehives (*Apis mellifera*); C) Honey production in Brazil, 2000–2015.** Data adapted from the United Nations Food and Agriculture Organization [12]. FAOSTAT search query: Pesticide Use (**item**: *insecticides+Total*, **elements**: *use*), Livestock Primary (**item**: *honey*; **elements**: *production quantity*) and Live Animals (**item**: *beehives*, **elements**: *stocks*).

We then conducted a historical analysis of the three variables for Brazil only, using data from 2000–2015. In this analysis, the response variables were (1) consumption of active ingredients of insecticides (Fig 5A), (2) number of bee hives (Fig 5B), and (3) honey production (Fig 5C). The predictive variable was the elapsed period, and the random effect was the years (longitudinal data).

Thus, we fitted a generalized linear mixed model (GLMM) to evaluate the response variables above (1 and 2) with a Poisson error distribution (link = log), considering they were count data. The link function (“log”) was chosen as a default, as it directly characterizes how a linear combination of predictors is related to prediction at the original scale. The first GLMM was evaluated with a quadratic term, while the second GLMM was evaluated with a cubic term. Both GLMMs were fitted using the *glmer* function of the “lme4” package [50]. The third variable (honey production) was investigated by fitting a linear mixed model (LME) with a quadratic term using the *lme* function of aforementioned package [50]. All final GLMMs and LMEs were selected after running some models with different polynomial regressions. Those with lower Akaike Information Criteria (AIC) were chosen (data not shown). The parameters and significance values of these models were extracted using the *Anova* function from the “car” package [51]. All analyses were carried out in the R statistical software environment [52].

## Results

### Brazil’s position in international surveys

Brazil is currently the world’s largest consumer of insecticides, at approximately 71,663 metric tons of active ingredients (Fig 1A), and has been among the greatest consumers over the last 15 years (S2 Table). According to currently available data, Brazil uses more insecticides than the second- through ninth-leading consumers combined (Fig 1A). However, when agricultural area is proportionally considered, we can see that insecticide use by Brazil is relatively low compared to other countries (Fig 1B).

However, when considering insecticide use vs. total size of agricultural area, we found a strong positive relationship (*F*_*(1*,*36)*_ = 953.08, *p* < 0.001, *R*^2^_adj_ = 0.962; Fig 2A). Similarly, if these data are reanalyzed without Brazil (since it is apparently an outlier), a positive relationship remains, but the percentage of variance explained is reduced (*F*_*(1*.*35)*_ = 24.83, *p* < 0.001, adj.*R*^2^ = 0.39, Fig 2B). When the total size of the agricultural area of these same countries is considered against its allocation to organic farming (i.e., non-traditional insecticide use), we did not find any relationship between these variables (*F*_*(1*,*32)*_ = 1.88, *p >* 0.05, *R*^2^_adj_ = 0.02, Fig 2C). However, when Brazil was removed from the sample due to its outlier status, we again found a positive relationship between total size of agricultural area vs. organic agricultural area (*F*_*(1*,*31)*_ = 11.31, *p <* 0.002, *R*^2^_adj_ = 0.24, Fig 2D).

On the other hand, Brazil (ranked 79^th^ in HDI worldwide) appears to be heading in the opposite direction when compared to the top-10 countries with the highest HDIs over the last 15 years (with the notable exception of Canada). In other words, whereas those countries have been reducing their demand for agrochemical supplies (insecticides) in their agricultural fields over recent years, Brazil has substantially increased its rate of consumption (Fig 3). In just the last 15 years, there was a 152% increase in use of insecticides in Brazil, with only a slight increase in the land area used for agriculture (approximately 8.1%).

Brazil (agricultural area: 282,589×10^3^ ha; beehives: 1,020,000) is also among the world’s leading honey producers, ranked 9th out of all countries (approximately 40,000 metric tons), although its production is 12 times less than that of the world leader, China (473,600 metric tons, agricultural area: 528,634×10^3^ ha; beehives: 9,131,487) (Fig 4A). However, Brazil has great potential for increasing its honey production once its agricultural area expands and especially because forage sources for honeybees remains underused (Fig 4B).

### Trends in Brazil’s use of insecticides, honey production and number of hives

The data show that over the last 15 years, Brazil has increased its use of insecticides in agriculture (Figs 3 and 5A), the number of hives in its apiaries (Fig 5B) and the volume of its honey production (Fig 5C). However, of these three variables, the greatest increase was in the use of insecticides on Brazilian crops. Consumption of the active ingredients of insecticides in Brazil more than doubled (152%) from 28,382 to the current level of 71,663 metric tons (quadratic, Poisson GLMM, degrees of freedom = 2, χ^2^ = 215.13, *p* < 0.0001, Fig 5A). Meanwhile, the number of bee hives increased by 23%, to approximately 1,020,000 hives (cubic, Poisson GLMM, degrees of freedom = 3, χ^2^ = 76.12, *p* < 0.0001; Fig 5B). Finally, honey production increased by 72%, to 380,000 metric tons (quadratic LME, F = 92.80, degrees of freedom = 2, *p* < 0.0001; Fig 5C).

## Discussion

The data indicate that, over the last 15 years, the world consumed more than five million metric tons of the active ingredients of insecticide and that Brazil has taken a leading role in this consumption among all the world’s countries (S1 Table). In recent years, Brazil has remained among the five highest insecticide consumers worldwide. Despite some asymmetry over country-reported information regarding insecticide use (in 2010, approximately 100 countries reported such information, versus only 40 countries in 2015; more details in S1 Table), Brazil has increased this consumption substantially. Thus, even though this nation has proportionally used less insecticide than other countries with smaller agricultural areas, the agrochemical consumption remains elevated, showing a bias for this pest control method despite alternatives that organic farmers use. The effectiveness of chemical products for controlling insect pests in agriculture is not a subject investigated in this study. We do not ignore that agrochemicals are useful [44]. However, their lethal and sublethal effects on non-target organisms can compromise the survival and viability of populations such as pollinators and parasitoid insects that are actually beneficial to agriculture [25,26,53].

Although the size of Brazil’s agricultural area is considerable, this only partly explains the need to use such large quantities of insecticides in the country’s agriculture over recent years. In contrast, alternative methods of pest control, such as integrated pest management (IPM), recombinant RNA technologies or even organic cultivation practices [45–47], are underused practices [21,47,54–56]. Farmers usually prioritize agrochemicals as a method of pest control, and many express reservations toward IPM, for example [21,54–56]. It should also be noted that farmers may be using agrochemicals incorrectly, not following the technical recommendations for their crops or the product recommendations (included in the information leaflets). If these infringements are indeed occurring, they could be impacting pollinators through excessive use of these products as a result of incorrect dosages, times of day, methods of application and even incorrect mixtures of different products [see 21]. For example, the Brazilian Ministry of the Environment recently published a document on risk assessment of insecticides regarding bees containing instructions and listing bee-friendly practices [57]. Therefore, even if farmers prefer to use insecticides for pest control, they can adopt attitudes that would reduce the risk of serious harm to bees [57].

Brazil has also shown itself to be a major global producer of honey, and this has increased over recent years. As the data show, Brazilian honey production has increased much more than the numbers of hives at the apiaries. The increase in honey production may be due to good public policies providing governmental support and technical training to beekeepers, such as teaching them how to better manage their hives and when to harvest honey from the combs. Even though Brazilian honey production is elevated, it could be greater if farmland was more efficiently managed in consort with beekeeping, since Brazil still has an extensive agricultural area that is both underused and little explored by both beekeepers and farmers. Moreover, such a partnership needs to proceed with caution; since 2010, the growth curve of both the number of hives and honey production in Brazil began to decrease again, while insecticide use continued to increase.

As such, considering that there have also been losses of hives because of the effect of insecticides [24], this creates a conflict of interest between the apiculture industry and agricultural farmers. In other words, these practices could make it difficult for apiaries to remain close to agricultural crops over the long term. These practices can also considerably reduce the benefits of the added economic value attributed to agricultural pollination provided by bees in Brazil [8–10], since the use of insecticides has increased much more (> 150%) than the number of hives (23%) and the volume of honey production (72%) in the country over the last 15 years.

This situation is alarming and needs to be addressed, as local research indicates that keepers of honey bees and stingless bees are reluctant to enter into partnerships with agricultural farmers because of excessive use of agrochemicals in the fields [11–13,24]. Considering the lethal and sublethal effects of insecticides on bees [27,32,58,59] and that a large proportion of the honey eaten worldwide, including in Brazil [41], contains insecticide residues, as does pollen [26,28], it would be unwise for Brazilian beekeepers to adopt partnerships with farmers before any deal.

Agricultural farmers and beekeepers should work in partnership, rather than avoiding each other, because both sides could improve the earnings from their production and increase the financial returns from their businesses. If insecticide use in Brazil could be reduced considerably, at least four major advantages of partnerships between farmers and beekeepers could be reaped: (1) greater growth of honey production, (2) increased profits for beekeepers from hive rental, (3) increased size of harvests in tonnage due to increased weight of fruit and seeds resulting from more effective pollination and (4) expansion and increased value of Brazilian agricultural products (“green card”) in more demanding international markets.

We recognize that agrochemicals can be used successfully to control pests. However, we also have alternative practices that are more friendly to pollinators, such as IPM (e.g., biological control, entomopathogens), crop rotation, recombinant RNA technologies, no-tillage systems, agroforestry and/or organic systems [45–49]. Nevertheless, farmers need to be financially aided by public policies and receive technical knowledge to implement all or most of these changes. Therefore, public agencies and private institutions should allocate more funding and offer technical training to help farmers reliably reduce insecticide use or replace it with other practices.

## Conclusion

Brazilian agriculture already benefits from pollination by wild bees from the few remaining small forest remnants surrounding cultivated areas [2,60]. However, this could be improved both by expanding the natural areas next to agricultural areas, minimizing the expansion of cultivation to fully take advantage of ecosystem services and managing hives within cultivated areas. More bees in the fields mean less insecticide on the crops. Agriculture and apiculture/meliponiculture are not incompatible businesses. In contrast, they are complementary activities that provide mutual environmental, social and economic value to each other. This issue cannot continue to be ignored. It is therefore suggested that the Brazilian public and private sectors rethink the prevailing model of harmful insect control and create incentives and an environment that stimulates favorable relationships between agricultural farmers and beekeepers in Brazil.

## Supporting information

S1 Fig Insecticides recently approved in Brazil that are toxic to bees. Ministério da Agricultura, Pecuária e Abastecimento [MAPA]. Sistema de Agrotóxicos Fitossanitários [AGROFIT]. In: AGROFIT. Consulta aberta [Internet]. Brasília/ Brazil; 2017. Available: http://agrofit.agricultura.gov.br/agrofit_cons/principal_agrofit_cons.
(PNG)

S1 Table Country-reported information on insecticide use, 2010–2015. **Are countries sending data about agrochemical use equally?.** United Nations Food and Agriculture Organization (http://www.fao.org/faostat/en/#data).
(XLSX)

S2 Table Worldwide insecticide use, 2010–2015. United Nations Food and Agriculture Organization (http://www.fao.org/faostat/en/#data).
(XLSX)

S3 Table Total agricultural area and total area allocated to organic agriculture in countries for which data about insecticide use are available. **Reference year, 2015.** United Nations Food and Agriculture Organization (http://www.fao.org/faostat/en/#data).
(XLSX)
