# Supplementary figures and images for: The dilemma of agricultural pollination in Brazil: Beekeeping growth and insecticide use
Source: PLoS One. 2018 Jul 6;13(7):e0200286. doi: 10.1371/journal.pone.0200286 (PMC6034858; doi:10.1371/journal.pone.0200286)

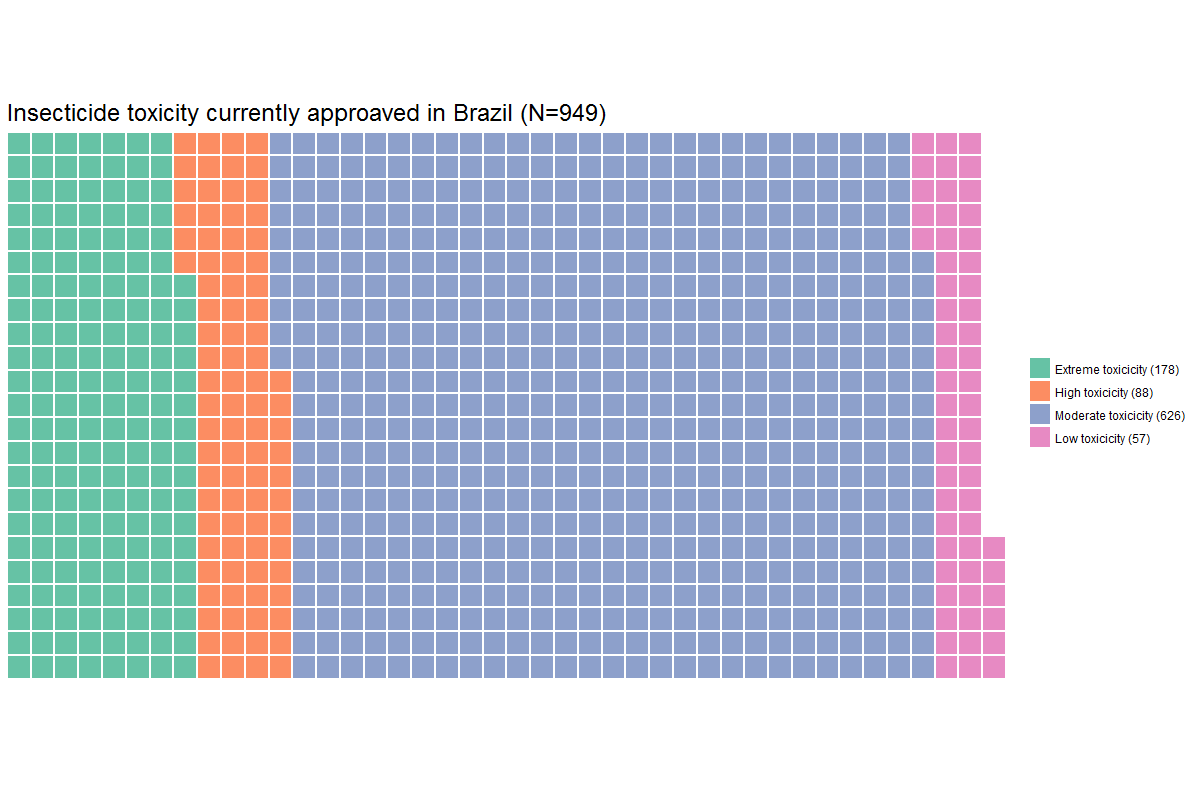

Supplement: S1 Fig — Ministério da Agricultura, Pecuária e Abastecimento [MAPA]. Sistema de Agrotóxicos Fitossanitários [AGROFIT]. In: AGROFIT. Consulta aberta [Internet]. Brasília/ Brazil; 2017. Available: http://agrofit.agricultura.gov.br/agrofit_cons/principal_agrofit_cons. (PNG) [file pone.0200286.s001.png]
